# Outpatient versus inpatient care for preterm premature rupture of membranes before 34 weeks of gestation

**DOI:** 10.1038/s41598-019-40585-8

**Published:** 2019-03-12

**Authors:** Hanane Bouchghoul, Gilles Kayem, Thomas Schmitz, Alexandra Benachi, Loïc Sentilhes, Chloé Dussaux, Marie-Victoire Senat

**Affiliations:** 1 Assistance Publique-Hôpitaux de Paris, Bicêtre Hospital, Department of Gynecology-Obstetrics, University Paris Saclay, Le Kremlin-Bicêtre, France; 2 Assistance Publique-Hôpitaux de Paris, Trousseau Hospital, Paris, Department of Gynecology-Obstetrics, France, University Pierre et Marie Curie, Paris, France; 3 Assistance Publique-Hôpitaux de Paris, Robert Debré Hospital, Department of Gynecology-Obstetrics, France, University Paris Diderot, Paris, France; 4 Assistance Publique-Hôpitaux de Paris, Béclère Hospital, Department of Gynecology-Obstetrics, University Paris Saclay, Clamart, France; 5 Department of Obstetrics and Gynecology, Bordeaux University Hospital, University Bordeaux, Bordeaux, France; 6 Assistance Publique-Hôpitaux de Paris, Louis Mourier Hospital, Department of Gynecology and Obstetrics, University Paris Diderot, Colombes, France

## Abstract

Preterm premature rupture of membranes (PPROM) is associated with an increased risk of serious maternal, fetal, and neonatal morbidities. We compared neonatal outcomes of women with PPROM before 34^+0^ weeks of gestation according to inpatient or outpatient management policy. 587 women with PPROM >48 hours, 246 (41.9%) in the group with an inpatient care policy (ICP) and 341 (58.1%) in the group with an outpatient care policy (OCP), were identified in France, from 2009 to 2012. Neonatal outcomes were compared between the two groups using logistic regression. A second analysis was performed to compare inpatient care and effective outpatient care (discharge from hospital) through propensity score matching. The outcome was a neonatal composite variable including one or more of the neonatal morbidity complications. The perinatal composite outcome was 14.6% with the ICP and 15.5% with the OCP (p = 0.76). After using the 1:1 ratio propensity score matching, effective outpatient care was not associated with a significantly higher risk of the perinatal composite outcome (OR 0.88, CI 0.35 to 2.25; p = 0.80) compared with inpatient care. Outpatient care is not associated with an increased rate of obstetric or neonatal complications and can be an alternative to hospital care for women with uncomplicated PPROM.

## Introduction

Preterm premature rupture of membranes (PPROM) before 34 weeks of gestation (WG) occurs in 1% of pregnancies^1–6^. PPROM is associated with an increased risk of serious maternal, fetal, and neonatal morbidities, especially the risks of preterm delivery, and infectious complications such as chorioamnionitis, sepsis and neonatal infection^1,7–10^. Expectant management in outpatient care may be appropriate in the absence of complication risk factors^11^ and improves women’s well-being and reduces health costs^12^. However, this practice is controversial because deliveries at home have been reported^13^. Moreover, obstetricians fear not managing fast enough serious complications such as home delivery, cord prolapse and placental abruption in an outpatient policy^14^.

There is currently no guideline concerning outpatient or inpatient management. In recent years, some centers have evaluated outpatient care management in PPROM and suggested that it is an acceptable option with comparable maternal and neonatal outcomes^14–17^. However, those retrospective studies had limitations, such as small sample size (18 to 61 patients) and comparison of care within the same center, which increases the risk of selection bias.

The Cochrane review on outpatient versus hospital care following PPROM includes two randomized trials with a total of 116 women^18^. It concluded that outpatient care is associated with fewer days in hospital and less cost. Both the American College^19^ and Royal College statements^20^ on PPROM highlight the lack of data to guide recommendations regarding hospital or outpatient care. As a comparison of different center policies has never been performed, we opted for this study design in order to limit the risk of bias.

Our aim was therefore to compare neonatal outcomes for women with PPROM before 34 WG, firstly according to their management policies - inpatient and outpatient care - and secondly between inpatients and effective outpatients (discharge from hospital).

## Material and Methods

### Study design, context

This retrospective study was carried out in six French tertiary care referral centers. Three centers had an inpatient care policy (ICP) (the maternity units of the Robert Debré, Intercommunal de Créteil and Angers Hospitals) and three centers had an outpatient care policy (OCP) (the maternity units of the Antoine Béclère, Bicêtre and Louis Mourier Hospitals). The study was conducted over a 4-year period between January 1, 2009 and December 31, 2012.

### Data sources

Women were identified through the electronic database of each center. Data were then collected from obstetric and neonatal hospitalization reports.

### Inclusion/exclusion criteria

Inclusion criteria were all patients admitted to hospital with a diagnosis of PPROM between 24^+0^ and 33^+6^ WG not delivered within the 48 hours following PPROM. Exclusion criteria were twin pregnancies, cases with fetal abnormalities conditioning prognosis and women who delivered in another maternity unit.

### Description of management in each group

Gestational age was determined using the routine ultrasound examination performed in the first trimester. In the OCP, criteria for outpatient care were absence of infectious signs (maternal fever (> = 38 °C), uterine tenderness, fetal tachycardia (>160 beats/min) and foul odor of amniotic fluid), signs of labor (regular uterine contractions, cervical dilatation over 3 cm), and patient acceptance.

A patient eligible for outpatient care was discharged from the second day of hospitalization with the following management: daily fetal cardiotocography, a laboratory blood sample twice a week and a weekly clinical and ultrasound exam. In the ICP group, the same protocol was applied, but the patient was not discharged until delivery.

All patients received betamethasone (two intramuscular doses of 12 mg 24 hours apart) for fetal lung maturation and antibiotic prophylaxis at PPROM (ampicillin or cefixime) according to the unit’s protocol. Antibiotic therapy was amoxicillin in five centers and cefotaxime in one center. The administration of tocolysis for a duration of 48 hours was not systematic and depended on the center’s practices. All patients were managed expectantly until spontaneous labor, signs of chorioamnionitis, fetal heart rate anomalies, or acute complications (placental abruption, cord prolapse). If not delivered at 37 WG, induction of labor or cesarean section was performed.

Maternal demographic data were collected: maternal age, weight, size, parity, history of PPROM, history of late miscarriage (abortion between 14^+6^ and 23^+6^ WG) or prematurity, previous cesarean section, cervical cerclage, and pregnancy pathology (abnormal placental insertion such as placenta previa, intrauterine growth restriction or gestational diabetes mellitus).

Data upon admission were collected: gestational age at PPROM, vaginal bacterial culture, maternal serum levels of inflammatory markers (C-reactive protein and white blood cell count^21^), fetal presentation, and amount of amniotic fluid evaluated by the single deepest pocket. The cervix was not systematically measured by ultrasound and this was left to the clinician’s discretion. In centers reporting an OCP, inpatient or outpatient care following the initial period in hospital and duration of hospitalization were collected.

#### Outcomes

The main variable of interest was a composite measure of neonatal outcome, including one or more of the following criteria: perinatal death, early neonatal infection with sepsis (defined as sepsis in a neonate with a positive blood or cerebrospinal culture in the first 48 hours of life), respiratory distress syndrome, bronchopulmonary dysplasia at discharge (oxygen requirement for a minimum of 28 days)^22^, grade 3 or 4 intraventricular hemorrhage^23^, and necrotizing enterocolitis^24^. Other neonatal outcomes included birth weight, gestational age at birth, and Apgar scores.

Other obstetric outcomes included gestational age at delivery, latency duration (i.e., the time interval between PPROM and delivery), chorioamnionitis, placental abruption, cord prolapse, and delivery mode. Chorioamnionitis was defined by the presence of maternal fever (> = 38 °C) with one of the following signs: uterine tenderness, fetal tachycardia (>160 beats/min) and foul odor of amniotic fluid^10^. Leukocytosis was defined as a white blood cell count above 15 G/L. Elevated CRP at PPROM was defined as above 15 mg/L. Maternal sepsis was defined by a suspected infection with at least two of the following criteria: a temperature above 38 °C or below 36 °C, a respiratory rate above 20 per minute (or PaCO_2_ < 32 mm Hg), a heart rate above 90 per minute, and a white blood cell count above 12,000 per mm^3^ or below 4,000 per mm^3^ ^25^. Post-partum endometritis was defined by the association of fever with foul lochia or a painful uterus at mobilization.

### Statistical analysis

Firstly, outcomes were compared according to the management policy of the center. The comparison of continuous variables was performed using Student’s t-test. Other variables were compared using the chi-square test and the Fisher exact test, where appropriate. Continuous variables are expressed as mean and standard deviation or median and interquartile range and categorical variables as percentages. All tests were two-sided at a significance level of 0.05. Neonatal morbidity and mortality were compared using a univariate logistic regression. Results were expressed as crude odds ratios (ORs) with their 95% confidence intervals (95% CIs), considering the inpatient policy group as the reference group.

Secondly, neonatal outcomes were compared between inpatients and effective outpatients using propensity score matching. Propensity score matching is a technique in which quasi-case/control pairs are produced from a retrospective cohort. In our study, propensity score is the probabilistic measure that reflects the propensity of the patient, based on other characteristics, to have outpatient management. For each group, the propensity to have outpatient management was calculated using multivariate logistic regression with outpatient as the dependent variable and all available patient characteristics as the independent variables. So, propensity score is used to reduce confounding variables and thus includes variables thought to be related to both outpatient management and outcome.

Effective outpatient was the actual discharge from hospital (because some patients were candidates for outpatient management but were not discharged from hospital for various reasons). This statistical strategy was used to minimize the effects of covariables related to differences in the two groups of patients^26^. Propensity score was constructed with the following variables: maternal age, ethnic group, smoking, BMI >25 kg/m^2^, a history of cesarean section, gestational age at PPROM, cervical length <25 mm at PPROM, anhydramnios at PPROM, administration of tocolysis, and cerclage during pregnancy.

Once the propensity score has been calculated for each observation, one must ensure that there is overlap in the range of propensity scores across inpatient and outpatient groups (called “common support”)^26^.

The analysis was based on propensity score matching with a 1:1 matching algorithm without replacement. The ORs for neonatal morbidity (early neonatal infection with sepsis, respiratory distress syndrome, bronchopulmonary dysplasia at discharge, grade 3 or 4 intraventricular hemorrhage, and necrotizing enterocolitis) and mortality were analyzed in the matched sample. Statistical analysis was performed using Stata 14 Software (StataCorp LP, College Station, TX, USA).

### Ethics

The study was approved by the Institutional Review Board (IRB, CEROG Comité d’éthique de la recherche en Obstétrique et Gynécologie) (protocol number 2018-OBS-0207). The need to obtain informed consent was waived by the ethics committee. An information sheet explained the purpose and the design of the study to the patients. The original identification of each patient in the database has been encrypted and replaced with surrogate identification. All methods were performed in accordance with the relevant guidelines and regulations.

### Details of ethics approval

The procedures of the study received ethical approval from the institutional review board (IRB, CEROG Comité d’éthique de la recherche en Obstétrique et Gynécologie) (protocol number 2018-OBS-0207).

## Results

**Figure 1:**
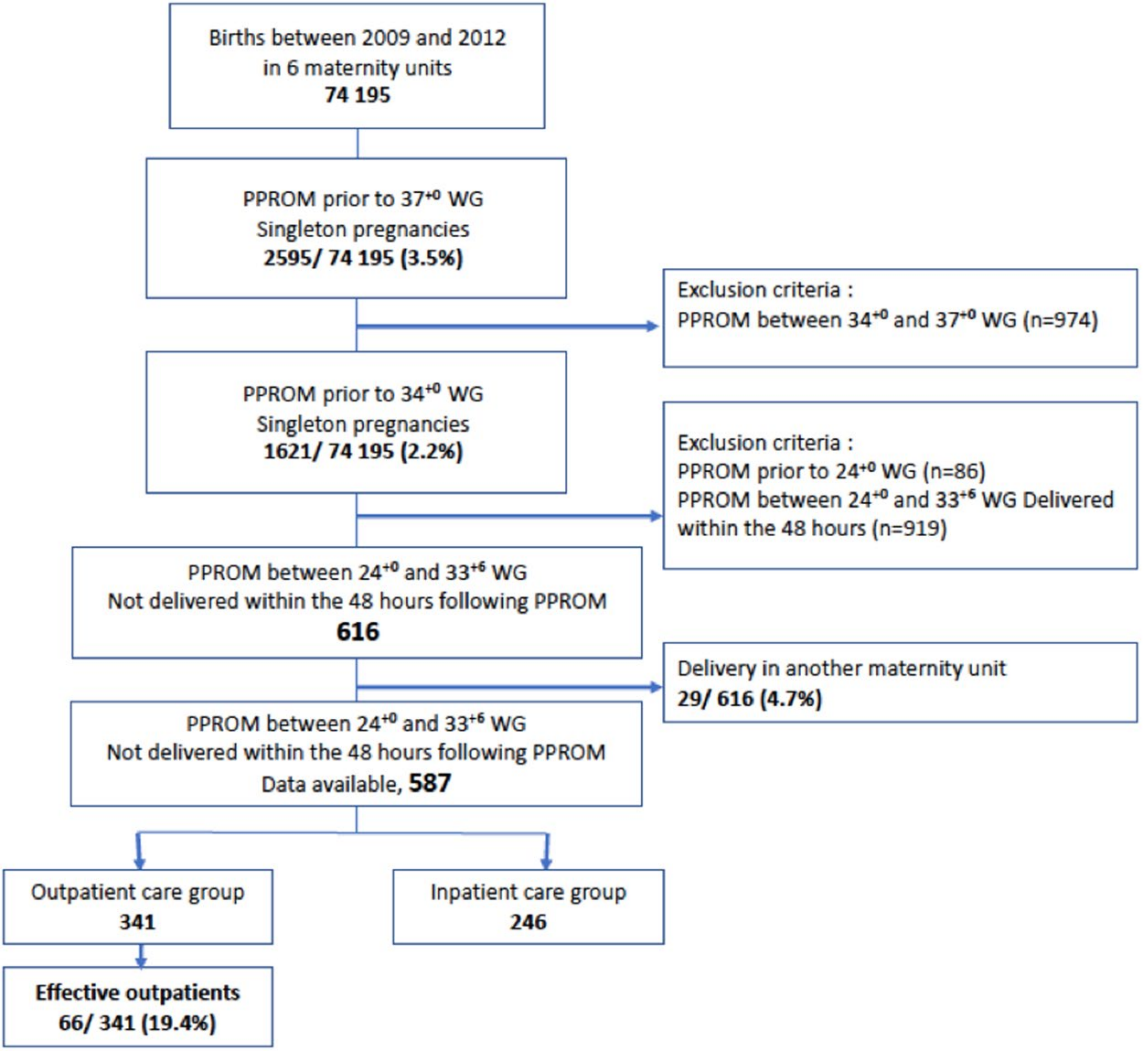
Flow chart.

During the study period, 74,195 patients were delivered in the six maternity units. The prevalence of PPROM among singleton births before 34^+0^ WG in the population was 2.2% (1621/74195), (Fig. 1). Six hundred sixteen patients with PPROM between 24^+0^ and 33^+6^ delivered 48 hours after PPROM. Twenty-nine patients (4.7%, 29/616) were excluded because they delivered in another maternity unit. Five hundred eighty-seven patients with PPROM between 24^+0^ and 33^+6^ WG were included, 246 in centers with an inpatient care policy (ICP) group and 341 of them in centers with an outpatient care policy (OCP) group. In the centers with outpatient care, the effective rate of outpatient was 19.4% (66/341 patients).

### First part: comparison according to the management policy of the center

Maternal demographic characteristics and obstetric history for women with PPROM between 24^+0^ and 33^+6^ WG in the ICP and OCP are presented in Table 1. There was no significant between-group difference except for parity (p = 0.04), with significantly more nulliparous women in the outpatient group. The median gestational age at PPROM did not differ between the two groups: 29^+2^ WG [interquartile range (IQR) 26^+3^–31^+4^] versus 29^+6^ WG (IQR 27^+2^–31^+6^; p = 0.14) in the ICP and OCP, respectively. No between-group difference was found for leukocytosis or CRP at admission (Table 2). The rate of sonographic anhydramnios and median cervical length was similar in the two groups.

**Table 1:** Demographic and obstetric characteristics in patients with PPROM between 24^+0^ and 33^+6^ weeks of gestation and delivered after 48 hours.

	Inpatient care policy n = 246	Outpatient care policy n = 341	p
Maternal age, years, median [interquartile range]	31^+2^ [26^+3^–35^+0^]	31^+0^ [27^+1^–35^+2^]	0.79
Nulliparous n (%)	90 (36.6%)	154 (45.2%)	0.04
Body mass index >25 kg/m^2^, n (%)	72/211 (34.1%)	106/297 (35.7%)	0.72
Smoking, n (%)	36/230 (15.6%)	33/300 (11.0%)	0.12
History of miscarriage after 14^+6^ WG and before 24^+0^ WG, n (%)	15 (6.1%)	23 (6.7%)	0.75
History of spontaneous preterm delivery after 24^+0^ WG and before 34^+0^ WG, n (%)	31 (12.6%)	31 (9.1%)	0.17
History of PPROM <37^+0^ WG, n (%)	14 (5.7%)	31 (9.1%)	0.13
Previous cesarean section, n (%)	42 (17.0%)	50 (14.7%)	0.43
Cervical cerclage, n (%)	25 (5.1%)	34 (5.0%)	0.82
Low-lying placenta and placenta previa, n (%)	6 (2.4%)	12 (3.5%)	0.45
Gestational diabetes, n (%)	16 (6.5%)	38 (11.1%)	0.06

PPROM: preterm premature rupture of membranes, WG: weeks of gestation.

**Table 2:** Comparison of management, delivery, obstetric and neonatal outcomes in patients with PPROM between 24^+0^ and 33^+6^ weeks of gestation and delivered after 48 hours.

Management	Inpatient care policy n = 246	Outpatient care policy n = 341	p
Gestational age at PPROM, weeks of gestation median [interquartile range]	29^+2^ [26^+3^–31^+4^]	29^+6^ [27^+2^–31^+6^]	0.14
Elevated CRP at PPROM (>15 mg/L)	45 (18.3%)	71 (20.8%)	0.45
Leukocytosis (>15 G/L)	56 (22.8%)	90 (26.4%)	0.31
Cervical length <25 mm	209/246 (46.9%)	221/341 (46.2%)	0.88
Cephalic presentation	171 (69.5%)	262 (76.8%)	0.05
Tocolysis n(%)	203 (82.5%)	193 (56.6%)	<0.001
Latency, days mean ± sd	15.2 ± 1.1	15.8 ± 0.8	0.68
Latency <7 days n(%)	91 (37.0%)	121 (35.5%)	0.71
Hospital length of stay,			
Initial, days mean + -sd	14.9 ± 0,5	11.4 ± 1.0	0.001
Duration >14 days, n(%)	86 (35.0%)	86 (25.2%)	0.01
Total duration of antibiotic therapy, days mean ± sd	6.2 ± 0.20	6.7 ± 0.25	0.10
**Delivery**			
Gestational age at delivery, weeks of gestation median [interquartile range]	31^+5^ [28^+6^–33^+6^]	32^+2^ [29^+5^–34^+0^]	0.09
Delivery 24–28 WG, n (%)	46 (18.9%)	44 (12.9%)	0.05
Delivery 28–32 WG, n (%)	82 (33.7%)	108 (31.7%)	0.60
Delivery >32 WG, n (%)	115 (47.3%)	189 (55.4%)	0.05
Labor induction, n (%)	66 (26.8%)	103 (30.2%)	0.37
Delivery mode			
Vaginal delivery, n(%)	170 (69.1%)	216 (63.3%)	0.15
Cesarean before labor, n (%)	51 (20,7%)	75 (22.0%)	0.71
Cesarean during labor, n (%)	25 (10.2%)	50 (14.7%)	0.11
Birth weight, g median [interquartile range]	1632 [1137–2000]	1790 [1330–2110]	0.04
Apgar score at 5 minutes <7, n (%)	21 (8.5%)	43 (12.6%)	0.12
**Obstetric outcome**			
***Neonatal***			
Composite perinatal outcome measure^*^	36 (14.6%)	53 (15.5%)	0.76
Neonatal sepsis^**^	9/243 (3.7%)	13/341 (3.8%)	0.95
***Prenatal***			
Chorioamnionitis^***^	41 (12.0%)	24 (9.8%)	0.39
Intrauterine death	3 (1.2%)	0 (0.0%)	0.18
Placental abruption	5 (2.0%)	9 (2.6%)	0.63
Cord prolapse	11 (4.5%)	5 (1.5%)	0.03
***Postnatal***			
Endometritis	10 (4.1%)	26 (7.6%)	0.08
Sepsis	2 (0.8%)	1 (0.3%)	0.38
Maternal hospital stay, days mean ± sd	4.4 ± 0.9	5.0 ± 0.16	0.01

PPROM: preterm premature rupture of membranes.

95% CI: 95% confidence interval; OR: odds ratio.

^*^Composite outcome measure of perinatal outcome comprises one or more of perinatal death, early neonatal infection with sepsis (defined as sepsis in a neonate with a positive blood or cerebrospinal culture in the first 48 hours of life), respiratory distress syndrome, bronchopulmonary dysplasia at discharge (oxygen requirement for a minimum of 28 days), grade 3 or 4 intraventricular hemorrhage, and necrotizing enterocolitis.

^**^Early neonatal sepsis was defined as a positive blood or cerebrospinal culture in the first 48 hours of life.

^***^Chorioamnionitis was defined as the presence of maternal fever (> = 38 °C) with one of the following signs: uterine tenderness, fetal tachycardia (>160 beats/min) and foul odor of amniotic fluid.

We also compared inpatients and outpatients among the OCP group for maternal and obstetrical characteristics (Table S1). Gestational age at PPROM was significantly lower for outpatients. Inpatients were more likely to have an elevated CRP (>15 mg/L) at PPROM, and a cervical length <25 mm.

Table 2 summarizes management and delivery characteristics. In the ICP, significantly more tocolytic treatment was administered than in centers with an OCP: 82.5% (203/246) versus 56.6% (193/341), respectively (p < 0.001).

Initial duration of hospitalization was significantly higher for ICP: 14.9 ± 0.5 days versus 11.4 ± 1.0 days in the OCP (p = 0.001). There was no significant between-group difference in latency duration, gestational age at birth, induction of labor, or delivery mode (Table 2).

However, the rate of births after 32 WG was lower in the ICP group, with 47.3% versus 55.4% in the OCP group (p = 0.05). The rate of births before 28 WG was higher in the ICP group, with 18.9% versus 12.9% in the OCP group (p = 0.05). The median birth weight was lower for neonates in the ICP group, with 1632 g versus 1790 g in the OCP group (p = 0.04).

There was no difference in the neonatal composite outcome between the two groups: 14.6% (36/246) versus 15.5% (53/341) in the ICP and OCP, respectively; p = 0.76. The chorioamnionitis rate was similar in the two groups: 12.0% (41/246) versus 9.8% (24/341) in the ICP and OCP groups, respectively; p = 0.39 (Table 2). There was no difference concerning intrauterine death, placental abruption, maternal sepsis, or endometritis rates between the two groups. The cord prolapse rate was significantly increased in the ICP group compared with the OCP group: 4.5% versus 1.5%, respectively (p = 0.03).

### Second part: comparison between inpatients and effective outpatients using propensity score matching

Propensity scores among the 66 patients in the effective outpatient care group and the 246 in the inpatient group were calculated. They take into account the possible criteria for in- or outpatient management. Gestational age at birth was not considered in the models because it can be a result of management.

Distribution of the propensity score before and after matching is displayed in Figs S1 and S2. Mean propensity score and covariates were balanced across in- and outpatients. Moreover, standardized differences in the weighted samples were less than 10% (Table S2). The matched groups were found to be well balanced for all recorded variables (Table S2). Thus, the quality of the matching was acceptable. The distribution of standardized differences after propensity scores matching is displayed in Figs S3 and S4. Each effective outpatient is matched only with control inpatients whose propensity score is within a predefined neighborhood of the propensity score of the outpatient. Therefore, 132 patients could be matched, with 66 in each group, inpatients and outpatients.

After propensity score matching, in the matched sample with a 1:1 ratio propensity score, the risk of neonatal morbidity assessed using the composite criteria among inpatients was comparable to the risk among effective outpatients (OR 0.88, 95% CI 0.35–2.25; p = 0.80) (Table 3). In addition, the risk of chorioamnionitis was comparable in the two groups (OR 0.54, 95% CI 0.15–1.95; p = 0.35) (Table 4). Risks of prolapse cord and placental abruption were no longer significantly different between the two groups.

**Table 3:** Neonatal outcomes in case of PPROM between 24^+0^ and 33^+6^ weeks of gestation according to outpatient or inpatient care, odds ratio before and after propensity score matching.

	Unmatched			Matched		
	Outpatient n = 66	Inpatient n = 246	p	Outpatient n = 66	Inpatient n = 66	p
Composite perinatal outcome measure^*^	0.92 [0.42–2.02]		0.84	0.88 [0.35–2.25]		0.80
Neonatal mortality	0.46 [0.06–3.68]		0.46	0.32 [0.03–3.22]		0.33
Neonatal sepsis ^**^	0.40 [0.05–3.21]		0.39	0.48 [0.43–5.48]		0.56
Respiratory distress syndrome	0.77 [0.41–1.44]		0.41	0.68 [0.31–1.50]		0.35
Bronchodysplasia at discharge	1.06 [0.34–3.35]		0.92	1.35 [0.28–6.58]		0.71
Necrotizing enterocolitis	0.66 [0.14–3.10]		0.60	0.49 [0.08–2.83]		0.42
Intraventricular hemorrhage	2.35 [0.82–6.72]		0.11	1.55 [0.40–5.99]		0.52

Propensity score was constructed with the following variables: maternal age, ethnic group, smoking, BMI >25 kg/m^2^, a history of cesarean section, gestational age at PPROM, cervical length <25 mm at PPROM, anhydramnios at PPROM, administration of tocolysis, and cerclage during pregnancy.

PPROM: preterm premature rupture of membranes; 95% CI: 95% confidence interval; OR: odds ratio.

^*^Composite outcome measure of perinatal outcome comprises one or more of perinatal death, early neonatal infection with sepsis (defined as sepsis in a neonate with a positive blood or cerebrospinal culture in the first 48 hours of life), respiratory distress syndrome, bronchopulmonary dysplasia at discharge (oxygen requirement for a minimum of 28 days), grade 3 or 4 intraventricular hemorrhage, and necrotizing enterocolitis.

^**^Early neonatal sepsis was defined as a positive blood or cerebrospinal culture in the first 48 hours of life.

**Table 4:** Maternal outcomes in the case of PPROM between 24^+0^ and 33^+6^ weeks of gestation according to effective outpatient or inpatient care, before and after propensity score matching.

	Unmatched				Matched		
	Outpatient n = 66	Inpatient n = 246		p	Outpatient n = 66	Inpatient n = 66	p
Chorioamnionitis^*^	0.60 [0.20–1.78]			0.36	0.54 [0.15–1.95]		0.35
Cesarean delivery^*^	0.40 [0.19–0.82]			0.01	0.33 [0.14–0.77]		0.01
Placental abruption^*^	0.40 [0.05–3.21]			0.39	1.00 [0.14–7.32]		1.00
Endometritis^*^	1.49 [0.45–4.91]			0.51	0.79 [0.20–3.10]		0.73
Latency, in days^**^	30.6 (19.0)		15.2 (16.7)	<0.001	30.6 (19.0)	25.4 (23.2)	0.16
Gestational age at delivery, weeks of gestation^**^	32^+3^ (3)		31^+2^ (3)	0.005	32^+4^ (3)	31^+5^ (4)	0.13
Post-partum hospital length of stay, in days^**^	4.3 (2.7)		4.4 (2.8)	0.77	4.3 (3.3)	4.8 (3.6)	0.27

Propensity score was constructed with the following variables: maternal age, ethnic group, smoking, BMI >25 kg/m^2^, a history of cesarean section, gestational age at PPROM, cervical length <25 mm at PPROM, anhydramnios at PPROM, administration of tocolysis, and cerclage during pregnancy.

PPROM: preterm premature rupture of membranes; 95% CI: 95% confidence interval; OR: odds ratio.

^ *^Odds ratio [95% CI]. ^**^Mean (standard deviation).

## Discussion

Our results show that an OCP in cases of PPROM between 24^+0^ and 33^+6^ is not associated with higher neonatal morbidity or mortality than an ICP. Severe acute complications such as placental abruption, cord prolapse or intrauterine death in centers were not more frequent with an outpatient policy. After propensity score matching, the risk of neonatal morbidity and neonatal sepsis was not increased for patients receiving outpatient care.

Three retrospective studies have shown that maternal outcomes (chorioamnionitis, mode of delivery) and neonatal outcomes (hospitalization in intensive care units, respiratory distress syndrome, intraventricular hemorrhage) were comparable between outpatients and inpatients, but these studies suffer from selection bias^14,27–29^. Indeed, most of the women in outpatient care were different from inpatients because of specific eligibility criteria with better prognostic factors. Our study compared inpatient to outpatient care using propensity score matching to equalize the women’s characteristics. Furthermore, two studies were conducted in a single center with small sample sizes^14,27^.

The Cochrane review reported data from two randomized trials with a total of 116 patients (55 patients in Carlan *et al*. and 61 in Ryan *et al*.)^18^. Inclusion criteria were singleton pregnancy, no signs of infection, amniotic fluid above 2 cm, cephalic presentation, cervical dilatation less than 4 cm, and resident in the country. These trials compared neonatal and maternal morbidity and perinatal mortality between planned outpatient and inpatient care in patients with PPROM before 37 WG. The risk of neonatal morbidity and mortality was not increased for outpatients. But the trials did not have sufficient statistical power to detect meaningful differences between inpatient and outpatient groups^18^.

The risk of acute complications such as placental abruption and cord prolapse was suggested as the main argument for the need for inpatient management^14^. Ellestad *et al*.^13^ assessed the outcome of pregnancies of patients hospitalized for PPROM who would have received outpatient care according to the criteria defined by Carlan *et al*.: PPROM before 37 WG, 48 to 72 hours of monitoring in hospital, without signs of infection or of the start of labor^16^. They concluded that because of the obstetric emergency or the premature birth of a child, being at home could have prevented adequate and sufficiently rapid care in a gestational age-appropriate center^13^. This is not in agreement with our results, which show that placental abruption rate was similar in the two groups and that the cord prolapse rate was significantly higher in the ICP. However, after propensity score matching, these differences were no longer significant.

One of the strengths of our study is that it relates to a large sample of 587 patients with PPROM with a non-selected population and a low rate of loss to follow-up (4.7%). The patients lost to follow-up gave birth in another maternity hospital, which could be the one where they were initially managed. The study design consisted of two complementary analyses. Firstly, we opted for an original study design with comparison of center policies. The center policy was applied to all patients in the centers even if effective outpatient care concerned only 19.4% of cases. Secondly, we compared effective outpatients with inpatients using propensity score matching. This strategy reduces selection bias due to nonrandom outpatient care assignment in the case of our observational data.

Our study, though, has several limitations. Its multicenter nature allowed for large enrollment but induced differences in management. Differences in protocol mainly involved the administration of tocolysis for centers with an inpatient policy, as well as the type of antibiotic therapy administered. However, those characteristics were taken into account by using the propensity score and, moreover, Combs *et al*. concluded that tocolysis longer than 48 hours did not increase latency or decrease neonatal complications compared with no tocolysis or tocolysis of less than 48 hours^30^. Lorthe *et al*. recently showed that tocolysis in cases of PPROM is not associated with improved obstetric or neonatal outcomes^31^. In addition, few patients were managed in outpatient care in the outpatient group (19.4%), which could have resulted in a lack of power. Our study was conducted between 2009 and 2012. Although obstetric management of PROM has not changed substantially since 2012, neonatal practice may have shifted since then, which may have implications for the contemporary applicability of the study’s findings. Finally, the retrospective nature of our study did not allow assessment of patient satisfaction and well-being, which are benefits of outpatient care^16^.

In our series, the observed rate of outpatients in centers declaring an outpatient policy was 19.4%, which is comparable to the rate of 11% to 17% reported in the literature^11,12^. Moreover, in the participant centers with an OCP, outpatient rates ranged from 14.8% to 30.9%, indicating variability in centers stating the same practices. This variability is multifactorial and is linked to the patient’s socioeconomic status, to the obstetrician, and to the center. Furthermore, patients can choose to refuse outpatient care management, after having received information from an obstetrician on PPROM, about risks, modalities of management and care provision. When the criteria for outpatient care management are not clearly defined, the outpatient decision remains subjective and is left to the discretion of the obstetrician. In addition, it should be noted that some obstetricians are reluctant to allow the discharge of these patients.

To answer the question of the comparison between these two management strategies, a randomized trial would be necessary, including an economic cost analysis and assessment of patient satisfaction. Due to the low prevalence of obstetric and neonatal complications, a very large number of subjects would be necessary and it would hardly be acceptable for patients to be selected randomly, which means that such a trial would be unrealistic. Moreover, current economic restrictions make it difficult to maintain these patients in conventional hospitalization in some centers. There is insufficient evidence to claim that outpatient management should currently be standard care for PPROM. For this purpose, we need more retrospective studies and a large national prospective study.

## Conclusion

Outpatient care remains an alternative to conventional inpatient care for PPROM as a result of prolonged care for patients who understand the issues involved.

## Supplementary information

Supplements

## Data Availability

The datasets analyzed during the current study are not publicly available due to patient privacy but are available from the corresponding author on reasonable request.
