# Supplementary material for: Outpatient versus inpatient care for preterm premature rupture of membranes before 34 weeks of gestation
Source: Sci Rep. 2019 Mar 12;9:4280. doi: 10.1038/s41598-019-40585-8 (PMC6414533; doi:10.1038/s41598-019-40585-8)

Outpatient versus inpatient care for preterm premature rupture of membranes before 34 weeks of gestation.

Supplements

Supplementary Table S1. Comparison of women characteristics at PPROM between in- and outpatients among the outpatient care policy group

	Inpatients n=275/341	Outpatients n= 66/341	p
Demographic and obstetric characteristics			
Maternal age, years, median [interquartile range]	31.2 [27.2-35.5]	30.1 [26.2-33.6]	0.15
Nulliparous n (%)	130 (47.3%)	24 (36.4%)	0.11
Body mass index > 25 kg/m^2, n (%)	88 (32.0%)	18 (27.3%)	0.46
Smoking, n (%)	24 (8.7%)	9 (13.6%)	0.23
History of miscarriage after 14^{+6} WG and before 24^{+0} WG, n (%)	20 (7.2%)	3 (4.5%)	0.43
History of spontaneous preterm delivery after 24^{+0} WG and before 34^{+0} WG, n (%)	24 (8.7%)	9 (13.6%)	0.63
History of PPROM < 37^{+0} WG, n (%)	26 (9.4%)	5 (7.5%)	0.63
Previous cesarean section, n (%)	41 (14.9%)	9 (13.6%)	0.79
Cervical cerclage, n (%)	31 (11.3%)	3 (4.5%)	0.10
Low-lying placenta and placenta previa, n (%)	11 (4.0%)	1 (1.5%)	0.32
Intra uterine growth retardation	22 (8.0%)	1 (1.5%)	0.06
Characteristics at PPROM			
Gestational age at PPROM	29^{+6} [27^{+4}-32^{+1}]	28^{+1} [26^{+1}-30^{+1}]	<0.001
Anhydramnios	41 (14.9%)	7 (10.6%)	0.37
Elevated CRP (>15mg/L) at PPROM	64 (23.3%)	7 (10.6%)	0.03
Leukocytosis	68 (24.0%)	16 (24.2%)	0.33
Cervical length <25mm	100 (36.4%)	14 (21.2%)	0.02

Supplementary Table S2. Covariates used to estimate the propensity score, standardized differences before and after matching

		Mean Outpatient	Mean Inpatient	Standardized Differences	p
**Maternal age (classes)**	Unmatched	1.97	1.99	-12.5	0.30
	Matched	1.97	1.97	0.0	1.0
**History of cesarean section**	Unmatched	13.6%	17.1%	-9.5	0.50
	Matched	13.6%	15.1%	-4.2	0.81
**Smoking**	Unmatched	13.6%	14.6%	-2.9	0.84
	Matched	13.6%	16.7%	-8.7	0.63
**BMI >25 kg/m^2**	Unmatched	27.3%	29.3%	-4.4	0.75
	Matched	27.3%	25.6%	3.3	0.84
**Ethnic group**	Unmatched	1.21	1.38	-29.6	0.04
	Matched	1.21	1.15	10.8	0.48
**Tocolysis**	Unmatched	53.0%	82.5%	-66.1	<0.001
	Matched	53.0%	56.1%	-6.8	0.73
**Gestational age at PPROM**	Unmatched	28.2	29.1	-35.7	0.01
	Matched	28.2	28.2	-1.9	0.91
**Cervical length <25 mm at the PPROM**	Unmatched	21.2%	41.1%	-43.7%	0.003
	Matched	21.2%	16.7%	10.0%	0.51
**Cerclage during pregnancy**	Unmatched	4.5%	13.8%	-26.9%	0.10
	Matched	4.5%	3.0%	4.4%	0.65
**Anhydramnios**	Unmatched	10.6%	20.7%	-28.0%	0.06
	Matched	10.6%	7.6%	8.4%	0.55

Supplementary Figure S1. Distribution of propensity scores before and after matching

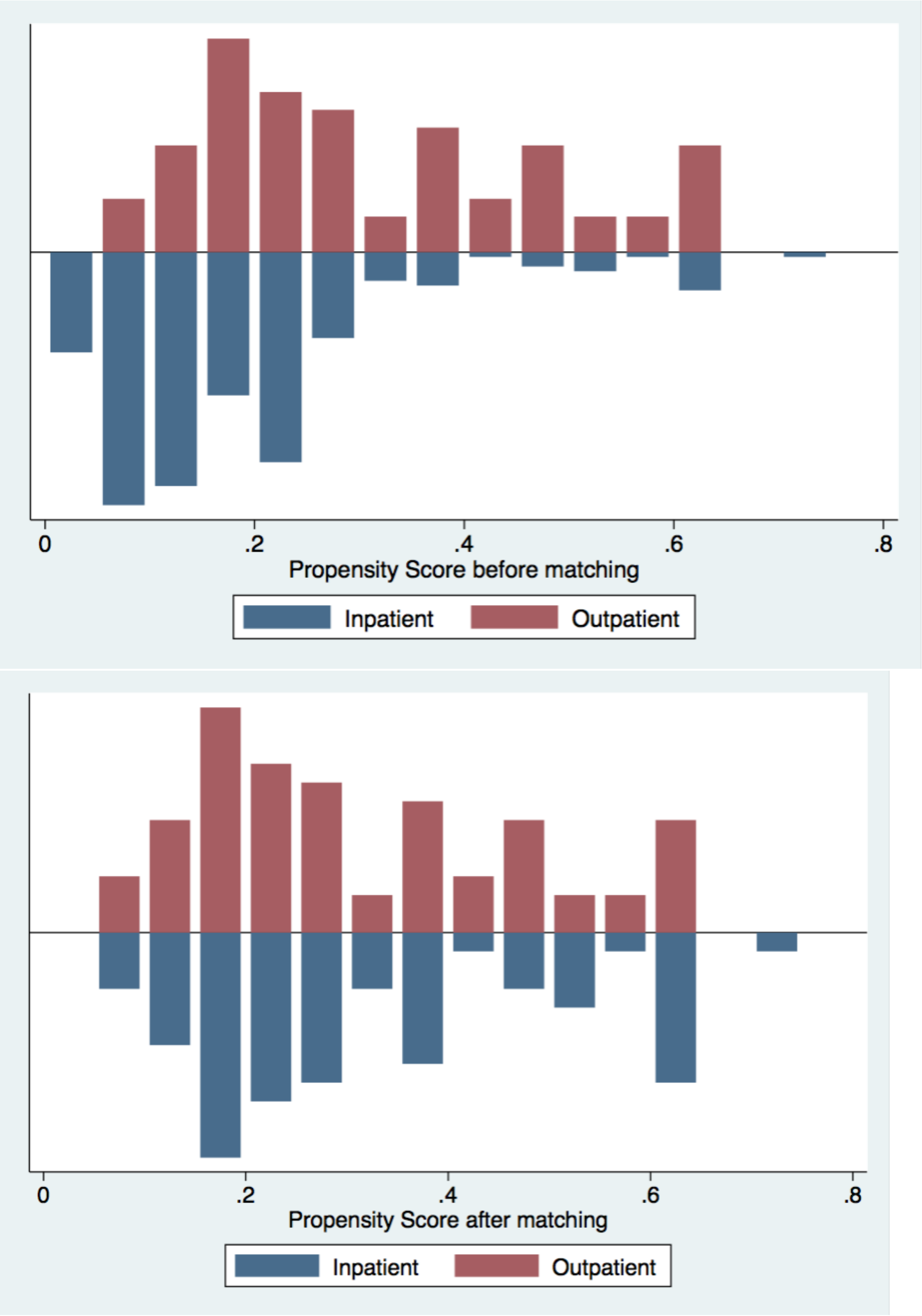

Supplementary Figure S2. Distribution of propensity scores before and after matching

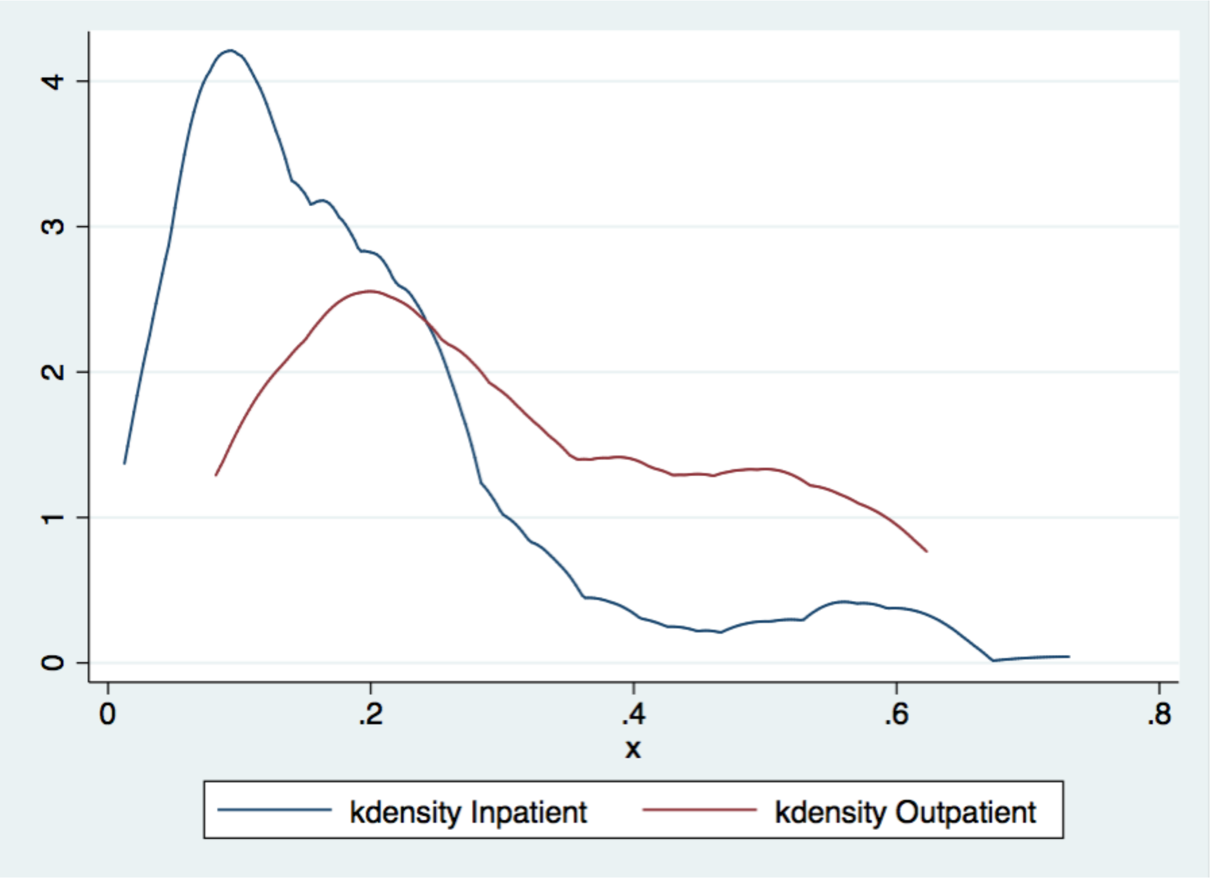

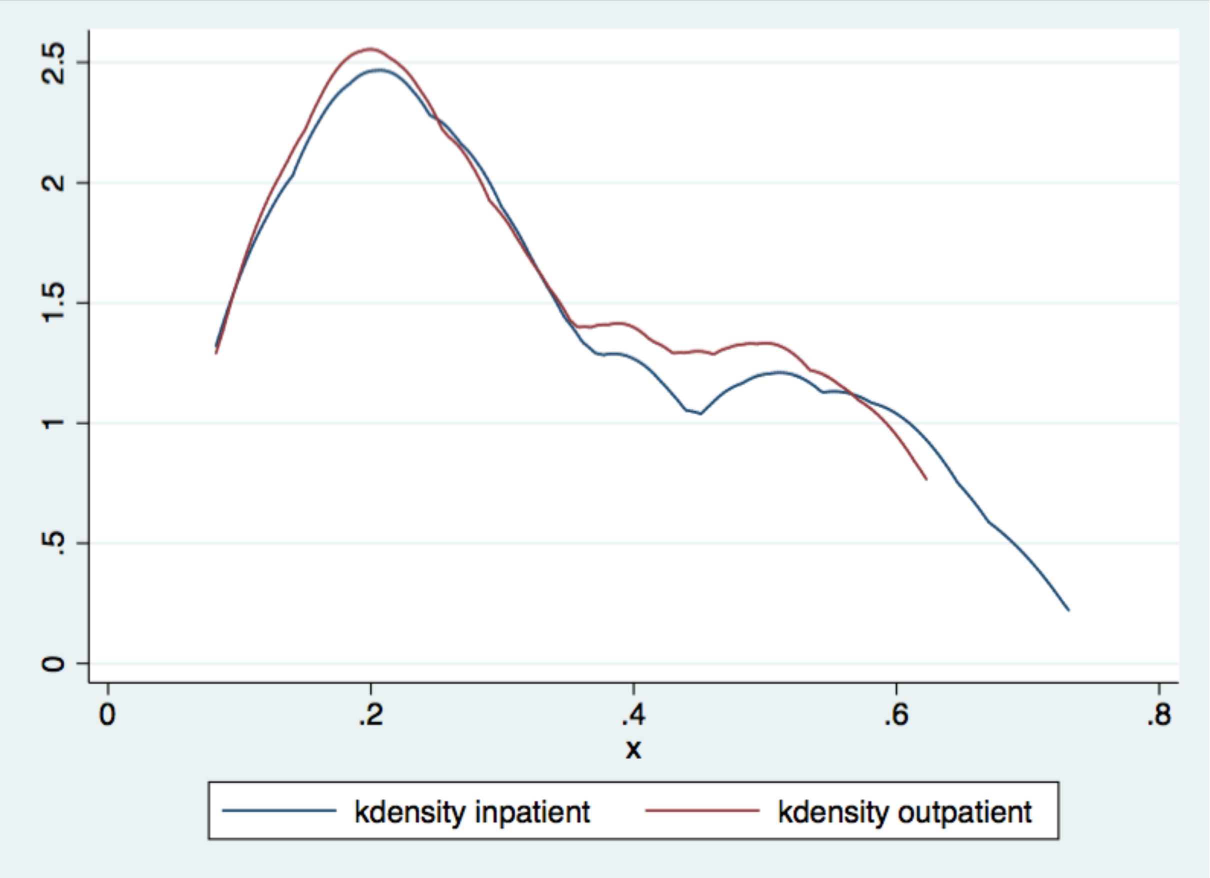

Supplementary Figure S3. Distribution of standardized differences after propensity scores matching

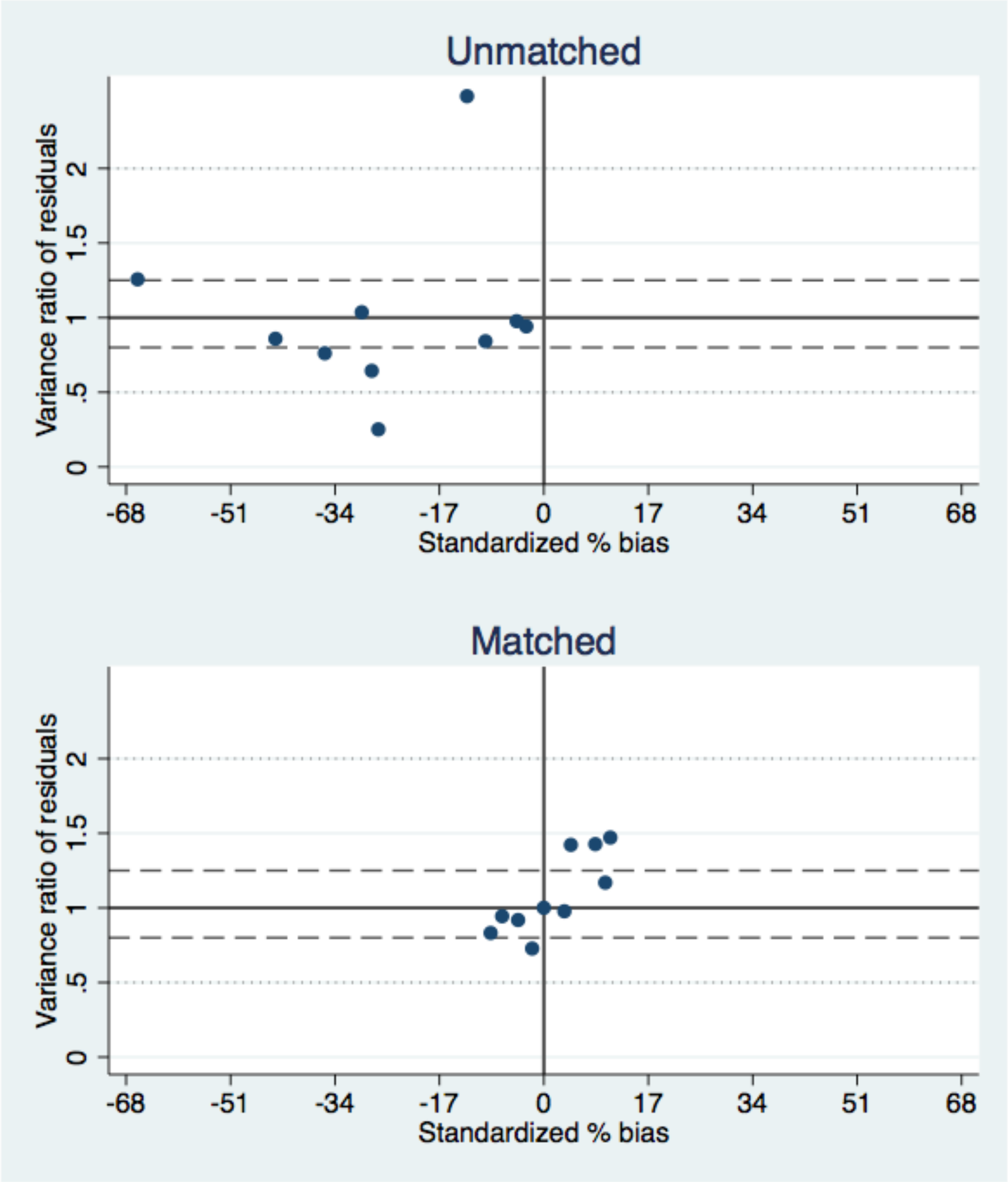

Outpatient versus inpatient care for preterm premature rupture of membranes before 34 weeks of gestation.

Supplementary Figure S4. Standardized differences in covariables before (blue) and after (red) matching on the propensity score

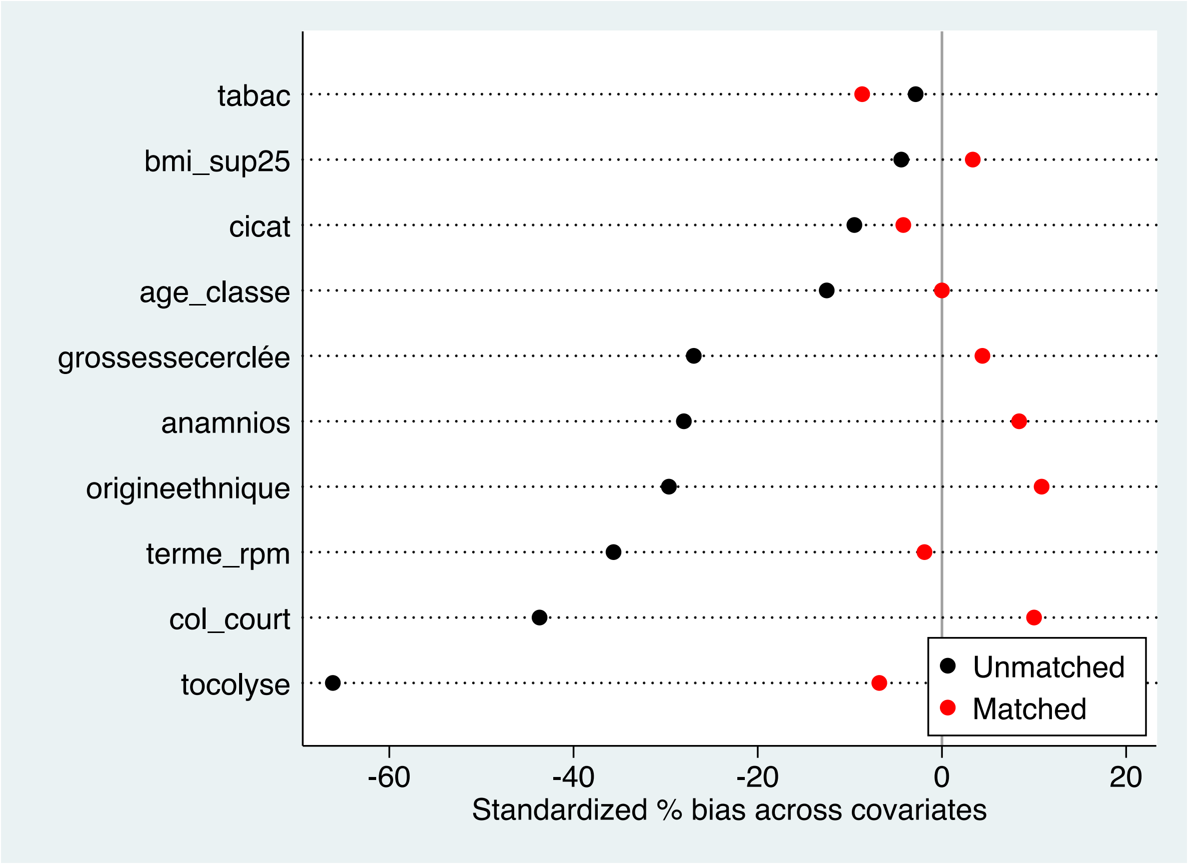

Supplement: Supplementary file 1 — Supplements [file 41598_2019_40585_MOESM1_ESM.pdf]
